# Weighted Sparseness-Based Anomaly Detection for Hyperspectral Imagery

**DOI:** 10.3390/s23042055

**Published:** 2023-02-11

**Authors:** Xing Lian, Erwei Zhao, Wei Zheng, Xiaodong Peng, Ang Li, Zheng Zhen, Yan Wen

**Affiliations:** 1 Key Laboratory of Electronics and Information Technology for Space System, National Space Science Center, Chinese Academy of Sciences, Beijing 100190, China; 2 School of Computer Science and Technology, University of Chinese Academy of Sciences, Beijing 100049, China; 3 Beijing Institute of Remote Sensing Equipment, Beijing 100190, China

**Keywords:** anomaly detection, sparse representation, matrix decomposition, weighted sparseness, adaptive threshold

## Abstract

Anomaly detection of hyperspectral remote sensing data has recently become more attractive in hyperspectral image processing. The low-rank and sparse matrix decomposition-based anomaly detection algorithm (LRaSMD) exhibits poor detection performance in complex scenes with multiple background edges and noise. Therefore, this study proposes a weighted sparse hyperspectral anomaly detection method. First, using the idea of matrix decomposition in mathematics, the original hyperspectral data matrix is reconstructed into three sub-matrices with low rank, small sparsity and representing noise, respectively. Second, to suppress the noise interference in the complex background, we employed the low-rank, background image as a reference, built a local spectral and spatial dictionary through the sliding window strategy, reconstructed the HSI pixels of the original data, and extracted the sparse coefficient. We proposed the sparse coefficient divergence evaluation index (SCDI) as a weighting factor to weight the sparse anomaly map to obtain a significant anomaly map to suppress the background edge, noise, and other residues caused by decomposition, and enhance the abnormal target. Finally, abnormal pixels are segmented based on the adaptive threshold. The experimental results demonstrate that, on a real-scene hyperspectral dataset with a complicated background, the proposed method outperforms the existing representative algorithms in terms of detection performance.

## 1. Introduction

Hyperspectral remote sensing images can provide an approximately continuous spectrum for each pixel owing to its hundreds of extremely narrow bands. It combines the traditional image features with the rich spectral features of ground objects, resulting in characteristics such as “hyperspectral resolution”, “spectrum integration”, “multiple spectral channels”, and “continuous imaging” [1,2,3]. It is highly well-liked in the fields of target detection and image classification as a result of these features [4,5,6,7]. Among them, image classification refers to assigning a class label to each pixel in the image and classifying it [8]. Target detection can be understood as a two-classification problem, which separates the target from the background [4]. Target detection can be divided into matching detection and anomaly detection according to whether the prior spectral information of the target needs to be used. As an unsupervised target detection technology, anomaly detection does not need a priori information concerning the target and separates the anomaly from the background [9]. Compared with background pixels, abnormal hyperspectral pixels typically exhibit two characteristics. From the perspective of spectral dimension, the spectral curve of anomalous pixels is entirely distinct from that of the surrounding background. From the spatial dimension, anomalous pixels occupied only a few pixels in the background. This also allows anomaly detection to detect anomalies that are significantly different from the surrounding background in terms of spectrum and to segment the target foreground and background through binary classification. Unfortunately, it is impossible to distinguish them. If the exception needs to be classified, the image classification method needs to be used to assign the class label to the exception pixel and classify the exception. A common method is to measure the distance between spectral features and anomalies to determine whether they belong to the same type [10]. In practical applications, because the spectral information of the target is difficult to obtain, anomaly detection is more practical in many scenarios [9]. Due to the diversity of target spectral information in matching detection and the complexity of atmospheric compensation in actual scenes, anomaly detection has received wide attention [11]. In recent years, many scholars globally have conducted in-depth research on hyperspectral anomaly detection. Hyperspectral anomaly detection technology has rapidly developed, and in addition to image classification, it is also widely used in mineral exploration [12,13], ground object classification [14], ecological monitoring [15,16], military reconnaissance [17,18], border monitoring and search and rescue [11] and many other fields.

Since the 1990s, hyperspectral anomaly detection has caught the fascination of researchers. Early anomaly detection systems were mostly guided by statistical modeling techniques. An anomaly detector named Reed Xiaoli (RX) [19,20,21,22], in which the background is assumed to conform to the multivariable normal distribution, has been proposed. It uses the samples in the scene to estimate the model parameters and judges whether it is an abnormal pixel by using the Mahalanobis distance to measure the difference of the abnormal pixel. According to the sample range selected for estimating the model parameters, RX can be used globally, also called global RX(GRX) [23], while it can also be used locally, called local RX(LRX) [23,24]. Then the kernel RX detector [25,26], which extends the linear low-dimensional non-Gaussian model to the Gaussian feature space that is high-dimensional and nonlinear, has been proposed. To improve the detection performance, it considers the correlation which is high-order and nonlinear between the hundreds of extremely narrow bands of HSI data. The clustering KRX (CKRX) [27] algorithm, which is based on clustering, can not only achieve considerable detection accuracy but also reduce the KRX’s computational complexity. It groups the background pixels and applies the algorithm which can achieve fast feature decomposition. The subspace RX (SSRX) [28] algorithm applies RX detection to the spectral segments of principal component analysis (PCA) [29]. The mathematical model of RX is simple and easy to handle, leading to its widespread usage. Only when the background model aligns with the assumed distribution of the detector, that is, it is a combination of Gaussian distributions, can the RX detector have a relatively good detection effect with low computational complexity [30]. However, in various applications, it is challenging to describe the complex background of a multivariate Gaussian distribution, and the model parameter estimation is polluted by the anomaly detection target.

Numerous algorithms have been proposed in order to achieve a more accurate background estimation. For instance, the Gaussian mixture-based anomaly detector (GMAD) [31] uses a group of weighted mixture models with unimodal Gaussian distribution to describe complex backgrounds. The cluster-based anomaly detector (CBAD) [32] employs clustering technology to classify hyperspectral images and uses RX detection in different categories. The collaborative representation-based detector (CRD) [33] sets internal and external windows around the pixel to be measured and assumes that the pixel between the internal and external windows is a background pixel; it determines whether the pixel is an abnormal pixel by checking if it can be represented by surrounding background pixel. The dual window-based eigen separation transform (DWEST) [34,35] hypothesis that the target pixel is located in the feature space of the difference between the covariance of the inner and outer windows. However, RX-based methods cannot overcome the limitations of Gaussian statistical distribution model assumptions. Covariance-based methods are insensitive to subtle differences between different categories within the local spectral ranges, and their accuracy is low. In recent years, compressed sensing has become popular. A new type of anomaly detector which is based on sparse representation was proposed [36,37,38]. Its aim is to reconstruct the measured pixels and calculate the reconstruction error using the learned background dictionary. Abnormal pixels had large reconstruction errors. Additionally, certain matrix decomposition theory-based anomaly detection techniques have been put forward. These algorithms assume that the background has low-rank features and a low probability of occurrence of anomalous pixels, with sparse features. Among them, the more representative algorithms are robust principal component analysis (RPCA) [39] and low-rank and sparse matrix decomposition (LRaSMD) [40,41,42,43]. However, when the background is complex, several edges and noise become sparse, leading to poor detection performance.

In addition, in recent years, because deep learning has a strong ability to capture depth features, it is also popular in hyperspectral image applications, such as image classification [44,45] and target detection [46]. For example, a spectral, spatial anomaly detection method based on hyperspectral band selection proposed by Xie et al. uses potential depth features to train unsupervised networks [47]. An anomaly detection method based on the depth convolution model was proposed by Mihai et al., and the model was learned through the self-supervised paradigm [48]. In order to further consider the local internal structure in remote sensing images. A kind of manifold-constrained AE network (MC-AEN)—based anonymous detector was proposed by Lu et al. [49]. However, the methods based on deep learning require a large number of data samples for supervision training. Unfortunately, in many practical application scenarios, the data samples are generally very limited.

Therefore, this paper proposes a weighted sparseness anomaly-based hyperspectral anomaly target detection method. Even in real hyperspectral datasets which have complex backgrounds, it can accurately identify the anomalies. The following are the study’s primary contributions:

(1) Traditional algorithms based on sparse representation build dictionaries using original hyperspectral images. The noise and sparse components in the original images significantly affected the detection performance. In this study, the WSA method uses the decomposed low-rank back to build a dictionary, which can effectively suppress the noise and interference caused by sparse components.

(2) Traditional matrix decomposition algorithms detect anomalies based only on the sparse matrix decomposed from the matrix, which is affected by non-abnormal pixels with large sparse parameters contained in the sparse component. The sparse coefficient divergence evaluation index (SCDI) proposed in this study can effectively suppress and enhance this target.

(3) The proposed method uses the abnormal information of low-rank and sparse components simultaneously, as well as it considers the sparse differences of space and spectrum segments, and utilizes the “space spectrum integration” feature of hyperspectral images, which significantly improves the detection performance. To the best of our knowledge, almost no anomaly detectors will simultaneously exploit the low-rank and sparse features embedded in hyperspectral remote sensing images.

The rest of this article is composed of four parts. In Section 2, the process of how to perform low-rank sparse reconstruction of hyperspectral images and obtain low-rank components as well as sparse anomaly maps is described in detail. Section 3 gives a very detailed description of how to calculate the sparse anomaly weighting factor and how to perform the threshold segmentation after weighting. Section 4 focuses on the description and discussion of the experiments, and we have selected some classical representative methods for comparison and seriousness of two commonly used hyperspectral practical scenarios. Finally, Section 5 summarizes the study.

## 2. Low-Rank and Sparse Decomposition

The anomalous pixels are distinct from the background pixels in the hyperspectral remote-sensing image. Specifically, it has a low probability, small occupation space [50], and sparse characteristics. The background pixels have strong spectral correlation and spatial continuity. As a result, the components representing the background have low-rank features and can be represented linearly by the surrounding background pixels. Based on the different features described above, the hyperspectral image can be modeled as a sum of matrices with different features, as follows:

(1) X=B+S+G

where $X\in R^{m\times n}$ (m represents the total amount of the spectral bands in the HIS, and n represents how many pixels there are in the HIS), B is a matrix with low-rank properties to represent the background components, S is a matrix with sparse features containing anomalies, and G is a matrix representing noise.

Zhou et al. [51] proposed a typical algorithm named GoDec, and it is widely used to determine the optimal solution in Equation (1). Thanks to employing the bilateral random projection (BRP) instead of the traditional singular value decomposition algorithm, GoDec is an approximation algorithm with short time-consuming. When searching for the optimal solution, first we need to construct the following objective function, which is used to minimize the decomposition error. At the same time, to obtain the results that meet our requirements, we have to restrict B’s rank to keep it within a low threshold, and the same for the sparsity of S.

(2) $$\{\begin{array}{c} \underset{B,S}{\min}||X-B-{S||}_{F}^{2}\\ s.t.\;rank\left(B\right)\leq p,\;card\left(S\right)\leq qn \end{array}$$

where the function of p is to restrict the rank of the matrix B, defining the maximum of the rank, and q is used to reflect the sparsity of matrix S. We can convert the problem in Equation (2) into the following two subproblems, and solve them alternately until convergence:

(3) $$\{\begin{array}{c} B_{t}=arg\underset{rank\left(B\right)\leq p}{\min}||X-B-S_{t-1}{\left|\right|}_{F}^{2}\\ S_{t}=arg\underset{card\left(S\right)\leq q}{\min}||X-B_{t-1}-{S||}_{F}^{2} \end{array}$$

where t indicates the iterations number during the training process. Initially, t=0, $B_{t}=X$, and $S_{t}$ is a zero matrix. What’s more, the singular value hard thresholding of $X-S_{t-1}$, indicating that the first p singular vectors of $X-S_{t-1}$ is used to update $B_{t}$, and use entry-wise hard thresholding of $B_{t}$ to update S, which indicates that the first *q* elements with values ranging from large to small.

(4) $$\{\begin{array}{c} B_{t}=\overset{p}{\underset{i=1}{\sum }}\lambda_{i}U_{i}V_{i}^{T},\;svd\left(X-S_{t-1}\right)=U\wedge V^{T}\\ S_{t}=P_{\Omega }\left(X-B_{t}\right),\Omega :\left|{\left(X-B_{t}\right)}_{i,j\epsilon \Omega }\right|\neq 0\;and\;\geq \left|{\left(X-B_{t}\right)}_{i,j\epsilon \bar{\Omega }}\right|,\;\left|\Omega \right|\leq q,\;\bar{\Omega }=supp\left(S_{t-1}\right) \end{array}$$

where $\lambda_{i}$ is the i-th maximum singular value of $X-S_{t-1}$; Ω is the non-zero set of the first q maximum terms of $\left||\left(X-B_{t}\right)|\right|$; $P_{\Omega }\left(\cdot \right)$ represents the projection process to the set Ω. The algorithm is terminated when the decomposition error of $||X-B-{S||}_{F}^{2}$ converges to a local minimum.

## 3. Proposed Method

We can reconstruct a low-rank background component from a hyperspectral image containing sparse anomalies and also extract the sparse anomalies. The main idea behind the sparse coefficient divergence index weighting factor is based on the fact that it is difficult to approximately indicate the abnormal pixels by local background pixels in the spectral or spatial domain. The sliding window strategy was used to build a local spectral and spatial dictionary in the low-rank components, reconstruct the measured pixel in the initial HSI, and solve the sparse coefficient divergence index weighting factor of the pixel. The sparse coefficient divergence index weighting factor includes the spectral sparse coefficient divergence index weighting factor and spatial sparse coefficient divergence index weighting factor.

### 3.1. Spectral Sparsity Coefficient Divergence Index Weighting Factor

**Figure 1:**
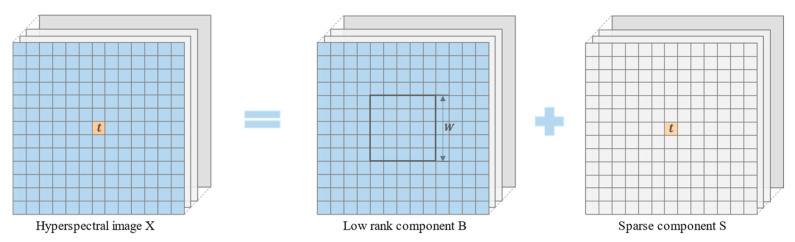
Low-rank sparse decomposition of hyperspectral data.

As shown in Figure 1, it is assumed that t is the measured pixel in the initial HSI X, and E is the corresponding local spectral dictionary, which is composed of all pixel spectra in the window, whose size is W, and the position of the measured pixel is the center in the low-rank component B. For the area near the boundary of the entire image, the image is expanded by mirroring.

If the measured pixel t belongs to the low-rank component, it is easy to find similar elements in E; thus, a group of compact sparse representation coefficients can be obtained. If t does not belong to the low-rank component, it will be difficult to find similar spectra in E for a sparse representation of the measured pixel t, because their spectral characteristics are not similar. The sparse representation of t is as follows.

(5) $$t\approx \alpha_{1}e_{1}+\alpha_{2}e_{2}+\cdot \cdot \cdot +\alpha_{\;N}e_{N}=\left[e_{1},e_{2},\ldots e_{N}\right]{\left[\alpha_{1},\alpha_{2},\ldots \alpha_{N}\right]}^{T}=E\alpha$$

where ${\left\{\alpha_{i}\right\}}_{i=1,2,\ldots N}$ is an unknown vector, each element corresponds to an element in E, denoting its abundance, and the value of N is W×W. When t is a target pixel, *α* exhibits a high degree of dispersion. An abnormal target can be distinguished from the background clutter signal by measuring the dispersion degree of α. Consequently, this study defined a sparse coefficient divergence index (SCDI) evaluation indicator that can effectively measure the dispersion degree of α. We employed this indicator as the weighting factor of the sparse anomaly map to enhance the target signal. The spectral sparse difference index weighting factor can be calculated as follows:

(6) $$SCDI_{spe}=\sqrt{\frac{\sum_{i=1}^{N}{\left(\alpha_{i}-\sum_{i=1}^{N}\alpha_{i}/N\right)}^{2}}{N}}$$

where N is the dimension of α.

### 3.2. Spatial Sparsity Coefficient Divergence Index Weighting Factor

**Figure 2:**
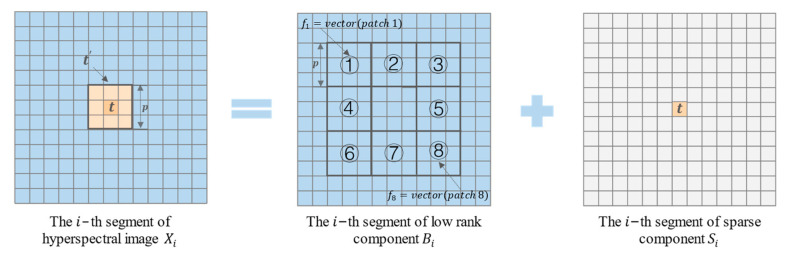
Schematic Diagram of Local Space Dictionary Construction. The numbers 1-8 are used to represent the eight neighborhoods of the pixel to be measured.

As shown in Figure 2, each spectral segment in the hyperspectral image can be handled as a common two-dimensional image. Assuming that t is the measured pixel in the initial hyperspectral image, on the initial hyperspectral data, a window with a size of p×p and the center pixel of t is used to replace the intensity characteristics of t and stretch the window block into a column vector $t^{\prime }$. Simultaneously, in the low-rank component, we assumed eight windows with a size of p×p around the center of the measured pixel, stretched the obtained window block into a column vector, and combined all these vectors into a new image block matrix as the local space dictionary F.

In space, the local space dictionary is used to represent the column vector $t^{\prime }$, as shown below:

(7) $$t^{\prime }\approx \beta_{1}f_{1}+\beta_{2}f_{2}+\cdot \cdot \cdot +\beta_{N}f_{N}=\left[f_{1},f_{2},\ldots f_{N}\right]{\left[\beta_{1},\beta_{2},\ldots \beta_{N}\right]}^{T}$$

where ${\left\{\beta_{i}\right\}}_{i=1,2,\ldots N}$ is an unknown vector. Similar to α, each item represents the abundance of the corresponding atom in F. N is eight. If t is from the target pixel, the obtained β will have a high degree of dispersion because we cannot represent the target pixel with the background pixel in a sparse way. Therefore, similar to the calculation method of the spectral sparse coefficient divergence index weighting factor, we can determine whether the pixel is the target pixel by measuring the dispersion degree of β. The weighting factor of the spatial sparsity coefficient divergence index of the i-th spectral segment is calculated as follows:

(8) $$SCDI_{spai}=\sqrt{\frac{\sum_{i=1}^{N}{\left(\beta_{i}-\sum_{i=1}^{N}\beta_{i}/N\right)}^{2}}{N}}$$

Assuming that there are M spectral segments in the hyperspectral image, the spatial sparsity coefficient divergence index weighting factor $SCDI_{spa}$ of the pixel to be measured can be expressed as

(9) $$SCDI_{spa}=\frac{1}{M}\overset{M}{\underset{m=1}{\sum }}SCDI_{spai}$$

Finally, the spectral sparse coefficient divergence index weighting factor and spatial sparse coefficient divergence index weighting factor are combined to form the sparse coefficient divergence index weighting factor. The formula used is as follows:

(10) $$SCDI=SCDI_{spe}+SCDI_{spa}$$

**Figure 3:**
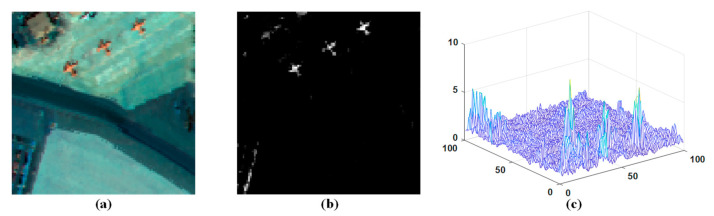
(**a**) San Diego scene; (**b**) Sparse difference index result image; (**c**) Three-dimensional display of sparse difference index results.

To better illustrate this point, Figure 3b shows the two-dimensional rendering of the spatial sparsity coefficient divergence index on the San Diego Airport dataset, and Figure 3c shows the spatial sparsity coefficient divergence index distribution. As the figure indicates, the SCDI of the majority of background pixels falls within a small threshold range, whereas the SCDI values of abnormal pixels and some edge pixels in the background are significantly larger. Therefore, the sparse difference index weighting factor can effectively strengthen the abnormal targets.

### 3.3. Anomalies Detection Based on Weighted Sparse Matrix

Through the low-rank sparse reconstruction of hyperspectral images, the low-rank matrix B, which represents the background component, the sparse matrix S which contains anomalies, and the noise matrix G are separated. Each row in the sparse matrix represents the sparse component of the spectral response for a single pixel. When the measured pixel is the target pixel, its sparse spectral response stands out significantly from the background pixel’s response. Assuming that the anomalies are randomly distributed in space, the row vectors of S are not related to each other; therefore, the Euclidean distance can be used to calculate the outlier value of each pixel $\;SA_{i}$ as follows:

(11) $$SA_{i}=\sqrt{\left(S_{i}-\bar{S}\right){\left(S_{i}-\bar{S}\right)}^{T}}$$

where $S_{i}\;$ refers to the i-th row vector in matrix S; $\bar{S}\;$ Represents the average row vector. If the value of $SA_{i}$ is large, the pixel has a high probability of being a target pixel. After obtaining the $SA_{i}$, the sparse weighting factor obtained above is used to weight the $SA_{i}$ to obtain the final anomaly detection operator $\delta_{wsa}$ as follows:

(12) $$\delta_{wsa}\left(x_{i}\right)=SCDI_{i}*SA_{i}$$

The anomaly detection operator yields a value exceeding the threshold *δ*, the pixel is deemed abnormal; otherwise, it is deemed to be a background pixel. All pixels in the entire image are traversed and the anomaly detection operator $\delta_{wsa}$ is calculated to achieve anomaly detection. Threshold δ is calculated as follows:

(13) δ=γ×Max+(1−γ)×Min 

where Max and Min refer to the highest and lowest values, respectively, of the anomaly-detection operators for all pixels. The value of *γ* is adjusted in combination with the application scenario which is restricted between 0 and 1.

The main algorithm process of WSA is summarized by Algorithm 1.
**Algorithm 1** WSA**Input:** (1) The original HSI data X;     (2) The restriction value of the rank p;    (3) The restriction value of the sparseness q;  Step 1. Solve the Equation (2) with Godec. Step 2. Construct the dictionary with matrix B, reconstruct initial HSI X, extract sparse coefficient, and calculate SCDI with Equations (6) and (8)–(10). Step 3. Calculate SA for each pixel with matrix S according to Equation (11). Step 4. Calculate the final anomaly detection operator $\delta_{wsa}$ with Equation (12). Step 5. Calculate the threshold value δ with Equation (13) and traverse the image. **Output:** Abnormal detection results.

In each iteration of GoDec, the computational complexity is less than $O\left[p\left(p^{2}+2NT+4N\right)\right]$ [51], the computational complexity of SA using Euclidean distance requires $O\left[NM^{2}\right]$, the complexity of SCDI requires O[N(W+M)], and the complexity of the final anomaly detection operator $\delta_{wsa}$ and finding the anomalies requires O[N]. Consequently, the computational complexity of WSA is $O\left[Tp^{3}+N\left(2Tp^{2}+4Tp+M^{2}+W+M+1\right)\right]$, where T is the number of iterations, p is the rank of the matrix, N and M are the number of pixels and spectral segments, and W is the size of the window.

## 4. Experimental Results

This section presents the results of five experiments we conducted on two classic HSI scenes to investigate the performance of the WSA anomaly detector.

### 4.1. Hyperspectral Data

To promote the diversity and dependability of the following experiment, we select two universal hyperspectral datasets with complex background scenes to implement the experiment. The backgrounds of these two scenes contain objects such as roads, bridges, and buildings and contain multiple anomalous targets. The abnormal targets in the image are characterized by a low probability of occurrence, small area and obvious spectral difference compared with the background [41], such as ships sailing at sea, aircraft parked at the airport and vehicles driving on the road. Table 1 shows information concerning these two datasets.

**Figure 4:**
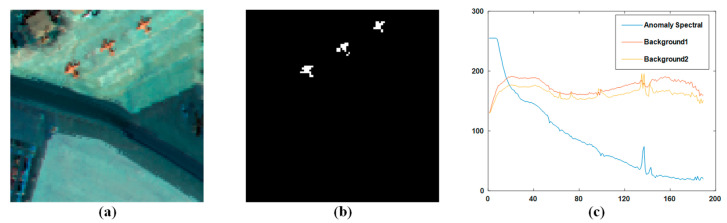
(**a**) San Diego scene; (**b**) Ground truth. (**c**) Spectra of main materials in the San Diego scene.

The following section provides additional and more thorough descriptions of these two datasets. The first dataset is the San Diego airport scene, and it is obtained by an airborne visible/infrared imaging spectrometer (AVIRIS) sensor. It has an initial dimension of 400 × 400 pixels, including 224 wavebands, and the spectral wavelength range was 370–2510 nm. In the experiment, since the original image is a little larger, we split a smaller subset from it. The removed subset contained 100 × 100 pixels. Considering bad bands, bands with low signal-to-noise ratio and water absorption area, the following bands (1–6, 33–35, 97, 107–113, 153–166, and 221–224) were removed, leaving only 189 effective bands, as shown in Figure 4a [9,41,42]. The three aircraft in the figure are considered as expected abnormal targets, and Figure 4b shows the corresponding real ground positions. The spectra of the main ground objects are shown in Figure 4c. We can see that the abnormal spectral curve is very different from the background.

**Figure 5:**
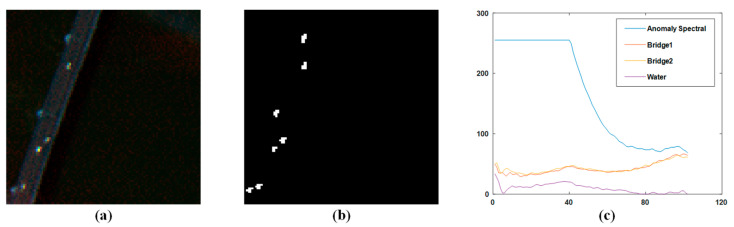
(**a**) PaviaC scene; (**b**) Ground truth. (**c**) Spectra of main materials in the PaviaC scene.

The second dataset is the PaviaC scene dataset. It is downloaded from the computational intelligence team at Basque Rural University. The dataset is obtained using a reflective optical system imaging spectrometer (ROSIS) sensor, and it accurately reflects the ground truth of the Pavia Center in northern Italy. The pixel size of the initial dataset is 1096 × 1096 pixels, 102 spectral bands and a spectral range of 430–860 nm. During the experiment, we divided a subset from the initial image into 100 × 100 pixels-sized pieces and selected 102 bands from the subset. In this scene, there are three ground objects, which are a bridge, water, and shadow. The bridge cars and the barren earth along the pier were considered abnormal pixels in the scene. Figure 5b shows the basic facts of the exceptions. The spectra of the main ground objects are shown in Figure 5c. From the figure, we can see that the anomaly has a spectral curve significantly different from the background.

### 4.2. Experimental Results and Discussion

#### 4.2.1. Effects from the Rank and Sparsity Level on Detection Performance

**Figure 6:**
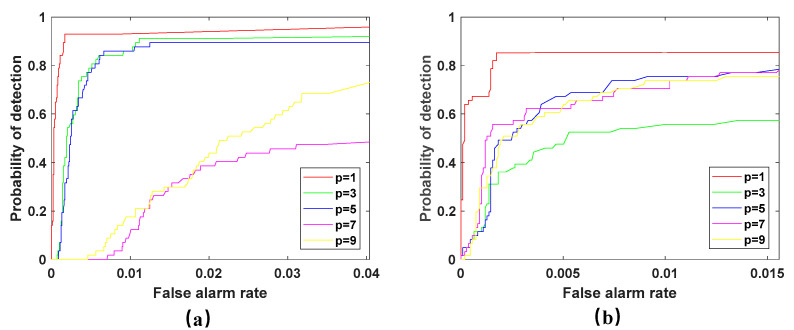
ROC curves of WSA detector with the changing background rank p. (**a**) San Diego scene; (**b**) PaviaC scene.

This part analyzes the influence of matrix decomposition coefficients p and q on the performance of the proposed method. In the first experiment, the value of the rank p of the background matrix in WSA on both two hyperspectral scenes is manually set between 1 and 9, and the step interval is 2. Set the sparsity level of the San Diego scene to 0.05 and the PaviaC scene to 0.15 through cross validation. As shown in Figure 6, the ROC curve of the WSA detector is shown on both two scenes with the value of background rank p from 1 to 9.

It can be seen from Figure 6 that in both scenes, when the rank is 1, the detection effect is the best, and with the increase of the rank, the detection performance is seriously affected, and the detection rate drops suddenly, especially in the PaviaC scene. It can be seen that the detector is very sensitive to the value of the parameter p, and the improper rank value will significantly affect the performance of the detector. If the value of rank is too high, the anomaly will be divided into low-rank components, and the detection performance will be reduced. For some more complex scenes, the rank cannot be too small, because it will lead to a lack of capture of the variability of the background spectrum, thus reducing the detection performance.

**Figure 7:**
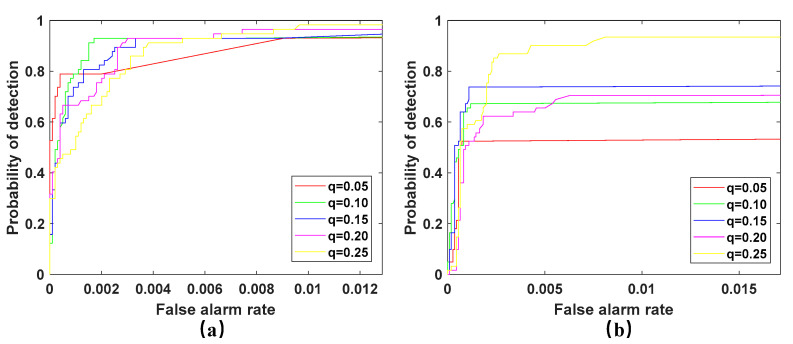
ROC curves of WSA detector with the changing sparsity level q. (**a**) San Diego scene; (**b**) PaviaC scene.

In the second experiment, the value of the sparsity level q  of the background matrix in the WSA on two hyperspectral scenes is manually set between 0.05 and 0.25, with a step interval of 0.05. Through cross validation, we set the rank p of the San Diego scene and PaviaC scene to one. As shown in Figure 7, the ROC curve of the WSA detector is displayed in two scenarios, with the value of sparsity level q ranging from 0.05 to 0.25.

It can be seen from Figure 7 that in the San Diego scene when q is 0.05, the detection effect is better, and when q is 0.1, the detection rate is also higher, but at the same time, the false alarm rate is also higher than when *q* is 0.05, and then with the increase of *q* value, the detection performance continues to decline. In the PaviaC scene, when the value of *q* is 0.15, the detection performance is the best. The detection performance first increases and then decreases with the increase of *q.* Therefore, the detector is also very sensitive to the value of parameter *q.* When the value of *q* is inappropriate, it will significantly affect the performance of the detector. This is mainly because, at first, with the increase of *q*, more anomalies are divided into the anomaly matrix, thus improving the performance. However, when the value of *q* is too high, it will cause the background or noise to be divided into the anomaly components, resulting in a decline in detection performance.

#### 4.2.2. Detection Performance of WSA

**Figure 8:**
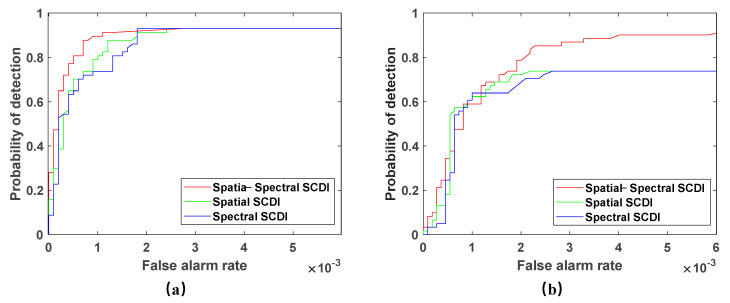
ROC curves of detection results of three weighting factors. (**a**) San Diego scene; (**b**) PaviaC scene.

We compare the detection results of the spatial spectral weighting coefficient with the spatial weighting coefficient and spectral weighting coefficient to illustrate the full use of the proposed spatial spectral weighting factor SCDI for the “spatial spectral integration” feature of hyperspectral images. As shown in Figure 8, in both scenarios, the detection results of the spatial spectrum weighting factor are significantly better than the other two.

**Figure 9:**
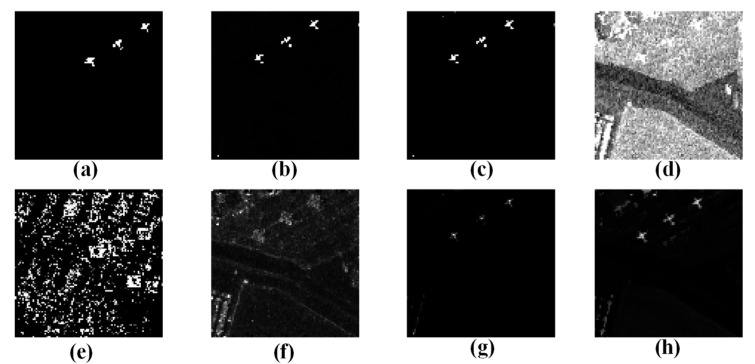
Results of the different approaches to San Diego scene; (**a**) Ground truth; (**b**) WSA; (**c**) LRaSMD; (**d**) RX; (**e**) LRX; (**f**) CRD; (**g**) FEBPAD; (**h**) RGAE.

**Figure 10:**
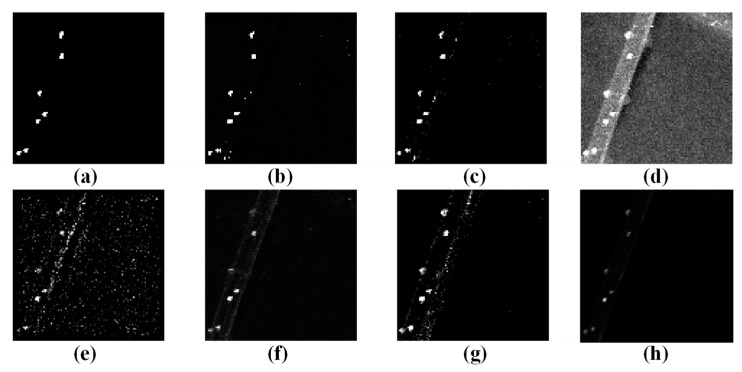
Results of the different approaches to PaviaC scene; (**a**) Ground truth; (**b**) WSA; (**c**) LRaSMD; (**d**) RX; (**e**) LRX; (**f**) CRD; (**g**) FEBPAD; (**h**) RGAE.

In order to assess the performance of the WSA detector in detecting anomalies, we compared it to six other algorithms commonly used for hyperspectral anomaly detection (LRaSMD [33,34], RX [18], LRX [19], CRD [26], FEBPAD [52], RGAE [53]). Our WSA detector and the six comparison detectors were applied to San Diego and PaviaC, and the results are displayed in Figure 9 and Figure 10, wherein, the abnormal targets can be effectively highlighted while the background is well suppressed with our proposed method and there are almost no or significantly few false alarms. However, RX, LRX, and CRD had poor detection effects, and LRX did not detect targets in the San Diego scene. In terms of background suppression, LRaSMD, CRD, FEBPAD and RGAE are relatively better than RX and LRX; however, they also have many false alarms, which are affected by edge information.

**Figure 11:**
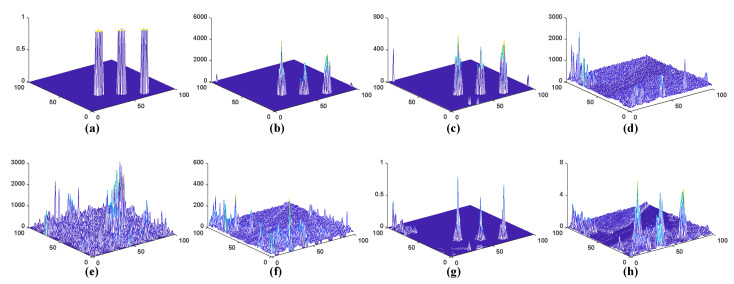
Plots of three-dimension detection outcomes of the multiple approaches to San Diego scene; (**a**) Ground truth (**b**) WSA; (**c**) LRaSMD; (**d**) RX; (**e**) LRX; (**f**) CRD; (**g**) FEBPAD; (**h**) RGAE.

**Figure 12:**
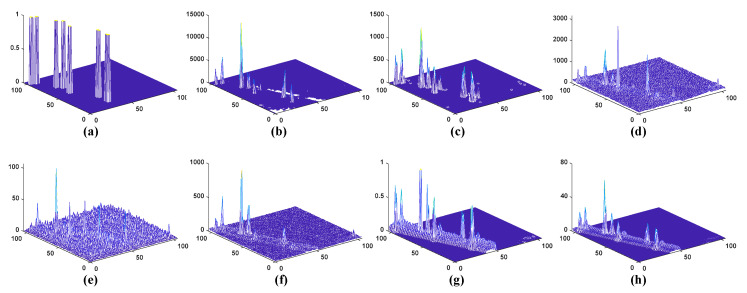
Plots of three-dimension detection outcomes of the multiple approaches to PaviaC scene; (**a**) Ground truth (**b**) WSA; (**c**) LRaSMD; (**d**) RX; (**e**) LRX; (**f**) CRD; (**g**) FEBPAD; (**h**) RGAE.

Figure 11 and Figure 12 present three-dimensional graphs of the detection outcomes of all seven algorithms on the two datasets without threshold segmentation. From the figure, we can observe that the WSA, LRaSMD and FEBPAD detectors can maintain the background pixels within a small range of values. However, compared to LRaSMD and FEBPAD, WSA can better suppress the background and enhance the target, and provide a clear separation between the target pixels and the background. In the detection results of the other three detection algorithms, there is a large fluctuation in the background pixels, which also makes distinguishing the target from the backdrop more challenging. In the San Diego scene, RX’s background pixel of the RX fluctuated the most. The LRX detection results reveal that the target and background are not separable. WSA outperforms the other six methods in terms of the detection effect. In the PaivaC scene, the background pixel of LRX fluctuates the most. The detection results of LRaSMD, CRD and FEBPAD were better than those of RX and LRX, but the background pixel also fluctuated slightly. The WSA and RGAE demonstrate superior separability compared to the LRaSMD, RX, LRX, CRD and FEBPAD detectors, as seen in the above observations.

**Figure 13:**
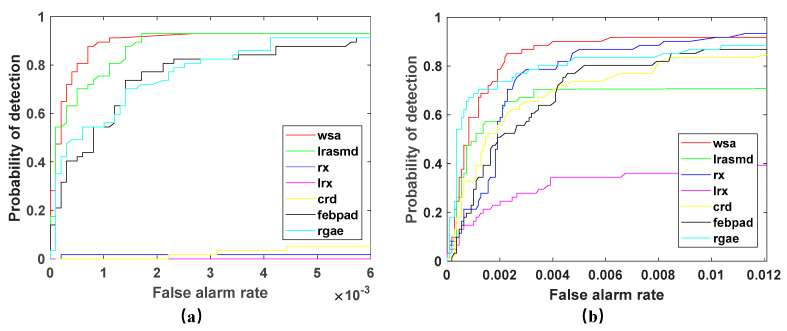
ROC curves of detection results of two scenes. (**a**) San Diego scene; (**b**) PaviaC scene.

To further illustrate the effectiveness of the WSA detector and evaluate the algorithm more accurately, Figure 13 displays the ROC curves of multiple detectors in two scenarios. From Figure 13, we can see that the WSA detector has the best detection performance in the San Diego scene, but in the PaviaC scene, the detection performance is lower than that of RAGE, but compared with RAGE, it can achieve a higher detection rate while having a lower false alarm rate. Therefore, in both test scenes, WSA has a very good ability to distinguish abnormal targets and has fewer error warnings.

In addition, Table 2 shows the calculation time required by the seven algorithms. All detectors were implemented in Matlab^®^2018b. The computer used in the experiment is Dell Precision 3551, which has a Xeon (R) W-10855M 2.81 GHz processor, 32 GB of RAM and a Windows 10 operating system. It can be seen from the table that, compared with other traditional methods, WSA has a longer running time, but WSA has excellent detection performance. Although the detection performance of RGAE in PaviaC scenarios is better than that of WSA, it has a much longer running time.

## 5. Conclusions

This study proposes a WSA detection algorithm for detecting hyperspectral image anomalies, which includes two parts: low-rank sparse reconstruction and spatial spectrum sparse coefficient divergence index weighting factor. The sparse component is weighted by a weighting factor to enhance the abnormal target and suppress the scene and noise to separate the target. Seven experiments were carried out on two real hyperspectral scenes to investigate the detection performance of the detector. Firstly, the influence of two main parameters, background, rank p and sparsity level q, on the performance of the detector is analyzed. Secondly, in order to prove that our algorithm makes full use of the advantages of “spatial spectrum integration” of hyperspectral images, a comparative experiment of the spatial weighting factor, spectral weighting factor and spatial spectral weighting factor is designed, and the results show that the detection effect of spatial spectral weighting factor is significantly better than the other two. Finally, compare the detection performance and running time of WSA with the other six methods. The detection performance of WSA ranks first in the San Diego scenario and second in the PaviaC scenario, but it can achieve a higher detection rate than RGAE with a lower false alarm rate. Compared with other traditional algorithms, WSA needs a long calculation time. However, compared with the RGCA which has the highest detection performance in the PaviaC scenario, the running time is much shorter. In future work, we will study the methods to improve the running speed of WSA, as well as how to detect point targets and how to use the detection results to classify different kinds of anomalies, so as to further optimize our detection algorithm.

## Figures and Tables

**Table 1:** Information of two datasets.

Scene	San Diego Scene	PaviaC Scene
Size	100×100	100×100
Number of spectral bands	189	102
Target Description	Three stationary parked aircraft.	The landscape of San Diego airport, with buildings and roads in the background, and there is no drastic change in spectra
Background Description	Exposed mounds of soil with no vegetation growth and the cars on the bridge	The background mainly contains rivers, bridges and shadows, and there is no drastic change in spectra

**Table 2:** Comparison of running time of seven detection methods.

Running Time of Detectors (s)							
Datasets	WSA	LRaSMD	RX	LRX	CRD	FEBPAD	RGAE
San Diego	45.3	15.5	1.2	30.2	3.7	3.5	87.3
PaviaC	40.1	11.2	1.1	15.3	3.5	3.2	65.5

## Data Availability

Not applicable.
